# Profile of Free Fatty Acids and Fractions of Phospholipids, Cholesterol Esters and Triglycerides in Serum of Obese Youth with and without Metabolic Syndrome

**DOI:** 10.3390/nu8020054

**Published:** 2016-02-15

**Authors:** Juliana Bermúdez-Cardona, Claudia Velásquez-Rodríguez

**Affiliations:** Research Group in Food and Human Nutrition, Universidad de Antioquia (UdeA), Calle 70 No. 52-21 Medellín 050010238, Colombia; juliana.bermudez@udea.edu.co

**Keywords:** obesity, metabolic syndrome, fatty acids, serum, youth

## Abstract

The study evaluated the profile of circulating fatty acids (FA) in obese youth with and without metabolic syndrome (MetS) to determine its association with nutritional status, lifestyle and metabolic variables. A cross-sectional study was conducted in 96 young people, divided into three groups: obese with MetS (OBMS), obese (OB) and appropriate weight (AW). FA profiles were quantified by gas chromatography; waist circumference (WC), fat folds, lipid profile, high-sensitivity C-reactive protein, glucose, insulin, the homeostasis model assessment (HOMA index), food intake and physical activity (PA) were assessed. The OBMS group had significantly greater total free fatty acids (FFAs), palmitic-16:0 in triglyceride (TG), palmitoleic-16:1*n*-7 in TG and phospholipid (PL); in the OB group, these FAs were higher than in the AW group. Dihomo-gamma-linolenic (DHGL-20:3*n*-6) was higher in the OBMS than the AW in PL and FFAs. Linoleic-18:2*n*-6 in TG and PL had the lowest proportion in the OBMS group. WC, PA, total FFA, linoleic-18:2*n*-6 in TG and DHGL-20:3*n*-6 in FFAs explained 62% of the HOMA value. The OB group presented some higher proportions of FA and biochemical values than the AW group. The OBMS had proportions of some FA in the TG, PL and FFA fractions that correlated with disturbances of MetS.

## 1. Introduction

Obesity is a pandemic that affects 34.3% of the adult population, generating an increase in the prevalence of chronic diseases [1]. Obesity is also present in youth; in 2013, the prevalence of overweight reached 22.6% in girls and 23.9% in boys in industrialized countries [2]. In Latin America, it is estimated that there are between 16.5 and 22.1 million obese adolescents, with a prevalence of overweight of 17% in Colombia, 20% in Brazil, and 35% in Mexico and Chile [3].

Obesity, mainly abdominal, induces metabolic disturbances, such as insulin resistance (IR), glucose intolerance, decreased high-density lipoproteins (HDL-C), elevated triglycerides (TG) and increased blood pressure, which together make up Metabolic Syndrome (MetS) [4].

In states of chronic excess energy, both subcutaneous and visceral adipocytes undergo hypertrophy and saturation of their storage capacity, along with infiltration and activation of macrophages; these events result in an increased release of fatty acids (FAs) and pro-inflammatory substances, such as tumor necrosis factor (TNF)α and interleukin (IL)-6, which generates tissue dysfunction and metabolic damage [5,6].

The release of pro-inflammatory substances and free FAs (FFAs) could be the connection between central obesity and metabolic disturbances. Once released into the portal circulation, FAs invade organs, such as the liver, where the excessive deposit of triglycerides affects lipoprotein synthesis and may induce simple steatosis or fatty liver (FL), which may be complicated by liver inflammation (non-alcoholic steatohepatitis, NASH), alterations that induce the development of non-alcoholic fatty liver disease (NAFLD), which is widely considered to be the liver expression of metabolic syndrome [7,8]. The FFAs also affect the pancreas where they impair insulin production (lipotoxicity); in addition, they simultaneously generate IR in muscle and adipose tissue through the activation of pathways such as protein kinase C (PKCθ) [6,9].

The FA profile can be modulated with food, as some FAs, such as linoleic-18:2*n*-6 and αlinolenic-18:3*n*-3, are not synthesized by humans and reflect dietary intake. Thus, circulating FA levels and cell membrane compositions depend on this intake. In serum, the FA profile specifically reflects the intake of the last two to six weeks, and tissue FAs represent the dietary patterns of months or years [10]. Blood levels of FAs, such as palmitoleic-16:1*n*-7 and DHGL-20:3*n*-6, are less dependent on intake and could reflect endogenous metabolism (lipogenesis) [11,12].

Research in adults has demonstrated the relationship between an altered FA profile, obesity, IR and MetS [13,14]. This profile is characterized by high percentages of saturated fatty acids (SFAs), such as palmitic-16:0 and stearic-18:0, and monounsaturated FAs (MUFAs), such as palmitoleic-16:1*n*-7, and decreased percentages of polyunsaturated fatty acids (PUFAs), including arachidonic acid (ARA-20:4*n*-6) and docosahexaenoic acid (DHA-22:6*n*-3) [14,15].

In obese individuals, a decrease in PUFAs may be due to a deficit in the synthesis and elongation of essential FAs, caused by metabolic disturbances arising from obesity, such as hyperinsulinemia; a high SFA intake and a low PUFA intake, especially omega-3 [16]. However, the FA profile in adults is affected by intervening variables, such as atheromatous disease, advanced metabolic disorders and long-term exposure to unfavorable lifestyle factors (*i.e.*, a sedentary lifestyle, overeating, and alcohol and tobacco consumption) [17].

In youth, describing the circulating FA profile could contribute to a better understanding of the obesity-IR-MetS relationship, given that confounding factors are less involved in metabolic analyses at early ages. However, the results in youth are also conflicting, possibly because fractions from which circulating FAs are recovered are different and not comparable among studies. Some obtain FA from cholesterol esters (CE) and others from phospholipids (PL), fractions in which FAs are mainly recovered from HDL-C and low-density lipoprotein (LDL-C), respectively, which are influenced by consumption [18]. Other studies recover FAs from TG, which is the fraction of highest proportion in circulation and mainly reflects endogenous synthesis of FAs produced from energetic nutrients (carbohydrates and fats) that travel in very-low-density lipoproteins (VLDL) [19]. Finally, others report FFAs released from adipose tissue and carried by albumin, which could be an indirect marker of the FA composition of adipose tissue in states of obesity if recovered after a 10- to 12-h fast [18,20]. Given the differences between fractions and modulations of the FA profile by diet, comparisons of results and the establishment of a consensus on FA profiles are complex. Therefore, the objective of this study is to evaluate the profile of circulating FAs in obese youth with and without MetS between 10 and 18 years old and to determine its association with nutritional status, lifestyle and components of MetS.

## 2. Materials and Methods

Study design: A cross-sectional study was developed that included 96 youth, boys and girls, 10 to 18 years old, selected from a previous population study [21]. 

The sample size necessary to form three groups was defined with the software Primer^®^, considering an alpha error of 0.05, a power of 85% and an expected minimum difference between the case and control groups in the concentrations of different FAs according to reports from other studies: Palmitic-16:0 in CE [22] and FFAs [23]; Palmitoleic-16:1*n*-7 in PL, CE and TG [22,24]; Stearic-18:0 in PL [22] and FFAs [23]; and DHGL-20:3*n*-6 in PL [24,25] and CE [22], which indicated a number of 32 youth per group. 

The groups were formed as follows: (1) obese with MetS (OBMS): body mass index (BMI) >98 percentile [26] and diagnosis of MetS; (2) obese (OB): BMI >98 percentile and without MetS; and (3) appropriate weight (AW): BMI between the 15–85 percentile and none of the components of MetS. Groups were matched one to one by age, gender, pubertal maturation and socioeconomic stratum. 

MetS was diagnosed when the affected youth had three or more of the following criteria: TG ≥110 mg/dL, HDL-C ≤40 mg/dL, fasting blood glucose ≥100 mg/dL, blood pressure ≥90 percentile and waist circumference (WC) ≥90 percentile [27].

Youth who consumed drugs for metabolic disturbances of MetS or nutritional supplements, who had diabetes mellitus (DM) II, who were elite athletes or who were pregnant or lactating were excluded from the study. 

Anthropometric evaluation: weight and height were measured; BMI was calculated (kg/m^2^) and was classified according to the 2007 World Health Organization (WHO) standard [26]; fat fold measurements were taken at the triceps and subscapular areas, % of body fat was calculated and classified according to Lohman [28]; and WC was taken and classified according to Fernández [29]. Measurements were performed with equipment and techniques for international use [28]. 

Food consumption: The 24-h recall was distributed on different weekdays. A second recall was consequently distributed among a random subsample constituted by 20% of the study population (19 adolescents) to calculate intra-individual variation [30,31]. The Evaluation Program of Dietary Intake (EVINDI-v4) was used [32]. The nutrient report was processed using the PC version of the Software for Intake Distribution Estimation (SIDE) program, Iowa State University, version 1.0, June 2004.

Physical activity (PA): The 3-day Physical Activity Recall (3DPAR) method was applied [33]. Metabolic equivalent (METs) values of each activity were obtained from the Compendium of Physical Activities of the American College of Sports Medicine [34]. The PA of 3–6 METs was classified as moderate to vigorous intensity (MVPA), and vigorous PA (VPA) was classified as >6 METs [35]. 

Time dedicated to watching television and playing video games: The reported times were converted into hours/day and were classified into two categories: less than three hours and three or more hours per day [36]. 

Clinical and biochemical tests: Pubertal maturation was evaluated and classified by self-report according to Tanner’s methodology [37,38]. 

Blood pressure was taken with a mercury sphygmomanometer (Riester^®^) and was classified according to Fourth Task Force methodology [39]. 

An antecubital venous blood sample was taken following a 10- to 12-h fast, and serum was obtained and stored at −80 °C. Total cholesterol (TC), HDL-C, LDL-C and TG were determined by spectrophotometry in an RA-50 photocolorimeter (Bayer, series 71663, Dublin, Ireland) using specific enzymatic colorimetric kits (BioSystems Reagents and Instruments, Barcelona, Spain). Blood glucose and insulin were determined by micro-particle enzyme immunoassay (MEIA) [40]. IR was estimated by the mathematical model of the HOMA (Homeostasis Model Assessment) index using the HOMA Calculator Version 2.2.2 software, copyrighted by the Diabetes Trials Unit of the University of Oxford. IR was defined as a HOMA ≥3.1, based on three criteria: 3.1 was the HOMA in the 95 percentile in the population under study [21], 3.1 was the cutoff point established by others authors [41,42]. hsCRP was determined by immunoturbidimetry and was classified as low cardiovascular risk <1 mg/L, average risk 1-3 mg/L and high risk >3 mg/L [43]. 

Profile of circulating FFAs: Lipid extraction from serum was performed according to the Folch method [44]. For separation of CE, TG and PL fractions, the methodology by solid-phase extraction (SPE) of Agren J [45] and Kaluzny M [46] was used. To the dry extract of each fraction, hexane and boron trifluoride (BF_3_) in 20% methanol were added, and the mixture was heated between 80 °C and 90 °C for one hour. [47]. Chromatographic analysis was performed in an Agilent 6890N gas chromatograph with a flame ionization detector (FID), TR-CN100 column, 60 m × 0.25 mm × 0.2 µm ID, oven temperature program beginning at 90 °C × 7 min, increased at a rate of 5 °C/min to 240 °C for 15 min, detector temperature of 300 °C, and He carrier gas at a flow of 1.1 mL/min. FA identification was performed by comparisons of retention times with the standard FAME Mix of 37 components (Supelco, Bellefonte, PA). The results are presented as relative amounts of each FA. 

Total concentrations of SFA, MUFA and PUFA were calculated from the sum of each FA from 14 to 22 carbons of each family, in each fraction. The product/precursor ratios were calculated as an indirect indicator of the activity of the desaturase enzymes. These ratios were as follows: stearoyl CoA desaturase = Palmitoleic-16:*1n*-7/Palmitic-16:0 and Oleic-18:*1n*-9/Stearic-18:0, delta-5 desaturase = ARA-20:4*n*-6/DHGL-20:3*n*-6 and delta-6 desaturase = DHGL-20:3*n*-6/Linoleic-18:2*n*-6, as previously reported [10,13,48]. 

Ethical management: The investigation was classified as having a minimum risk according to the Colombian Ministry of Health, Resolution 008439, Article 11 of October 1993. The study was approved by the University of Antioquia Bioethics Committee. Informed consent was signed by the youth and their parents and included the Helsinki declaration. 

Statistical analysis: Normality was established with the Shapiro-Wilk test. The difference among groups was performed by analysis of variance (ANOVA) or Kruskall-Wallis. For comparisons between two groups, Scheffe or Mann-Whitney U tests were performed. Qualitative variables were expressed with frequencies; quantitative variables, with means and standard deviations or medians and interquartile ranges. For associations of qualitative variables Chi^2^ and Odds Ratium were used, while Pearson *R* or Spearman *Rho* were used for quantitative variables. A stepwise multiple linear regression model was applied to explain the HOMA (dependent variable in logarithmic units) with WC, average METs, linoleic-18:2*n*-6 in TG, DHGL-20:3*n*-6 in FFAs and total FFAs as independent variables; the model was adjusted by food intake variables: daily intake of calories, simple carbohydrates, saturated fat, monounsaturated fat and polyunsaturated fat. *R^2^* and ANOVA determined the fit of the model, and fulfillment of assumptions (Durbin Watson, normality of residuals, inflation factor of the variance and independence) was checked. Finally, the β values of the crude and the adjusted model were transformed with antilogarithm to express in units of the dependent variable: HOMA, in Table 5. A *p* < 0.05 was considered significant. Statistical analysis was performed using the program Statistical Package for the Social Sciences (SPSS^®^) V 21.0. 

## 3. Results

Matched variables between groups did not show significant differences (Table 1).

**Table 1:** Matched characteristics of the study groups.

		OBMS *n* = 32	OB *n* = 32	AW *n* = 32	*p* *^,†^
Age (Me, IQR)		13.9 (4.8)	14.2 (4.1)	14.0 (4.2)	0.93 *
Gender (%)	Boys	56.3	56.3	56.3	1.00 ^†^
	Girls	43.8	43.8	43.8	
Pubertal Maturation (%)	Prepubescent	12.5	12.5	12.5	0.99 ^†^
	Pubescent	25.0	21.9	25.0	
	Post-pubescent	62.5	65.6	62.5	
Socioeconomic Stratum (%)	Low	53.1	53.1	50.0	0.84 ^†^
	Medium	37.5	31.3	38.5	
	High	9.4	15.6	11.5	

OBMS: obesity with metabolic syndrome; OB: obesity; AW: Appropriate weight; Me: medians; IQR: interquartile ranges; * Kruskal Wallis; ^†^ Pearson Chi squared.

Both obese groups showed similar behaviors for fat percentage and inflammation. The risk of mild chronic inflammation (high-sensitivity C-reactive protein (hsCRP > 1 mg/dL)) in obese youth was 2.6 times greater than in the appropriate weight (AW) group (odds ratio 2.6; confidence interval 1.27–5.54; *p* = 0.001), only this risk was significant; hsCRP correlated with fat percentage (*r* = 0.51 *p* = 0.001). The obese with MetS (OBMS) group had higher BMI, WC, blood glucose, insulin, HOMA and TG and lower HDL-C compared to the other two groups (*p* = 0.001). A total of 97.3% of the OBMS group simultaneously had high TG and low HDL-C, according to the criteria used in the diagnosis of MetS in this study. Of the OBMS, 53.1% simultaneously had high WC and HOMA; both variables were correlated (*Rho* = 0.71; *p* < 0.001) (Table 2).

**Table 2:** Anthropometric, biochemical, food intake and physical activity variables of the study groups ^1^.

	OBMS *n* = 32	OB *n* = 32	AW *n* = 32	*p* *^,†^
Anthropometric				
BMI kg/m^2^	31.1 ± 5.0 ^a, 2^	27.1 ± 2.9 ^b^	20.2 ± 2.3 ^c^	<0.001 *
Waist circumference cm	91.2 ± 10.9 ^a^	80.5 ± 5.6 ^b^	67.6 ± 5.4 ^c^	<0.001 *
Subscapular fold mm	29.9 ± 10.9 ^a^	25.5 ± 8.1 ^a^	11.5 ± 3.5 ^b^	<0.001 *
Triceps fold mm	25.2 ± 6.7 ^a^	23.6 ± 6.2 ^a^	12.9 ± 4.1 ^b^	<0.001 *
% fat	38.1 (18.7) ^a^	34.7 (15.8) ^a^	22.2 (14.2) ^b^	<0.001 ^†^
**Biochemical**				
Blood glucose mg/dL	90.3 ± 8.2 ^a^	83.7 ± 7.5 ^b^	83.3 ± 6.9 ^b^	<0.001 *
Insulin mU/L	25.7 (12.4) ^a^	11.5 (7.8) ^b^	7.1(4.1) ^c^	<0.001 ^†^
HOMA	3.2 (1.7) ^a^	1.5 (1.0) ^b^	0.9(0.5) ^c^	<0.001 ^†^
Cholesterol (mg/dL)	192.6 ± 50.3 ^a^	172.3 ± 34.5	162.1 ± 27.9 ^b^	<0.001 *
Triglycerides (mg/dL)	162 (94) ^a^	92 (37) ^b^	71 (32) ^c^	<0.001 ^†^
HDL-C (mg/dL)	38 (6) ^a^	45 (15) ^b^	54 (15) ^c^	<0.001 ^†^
LDL-C (mg/dL)	99 (61) ^a^	88 (37) ^a^	79 (31) ^b^	<0.001 ^†^
hsCRP (mg/dL)	1.7 (2.9) ^a^	0.9 (2.5) ^a^	0.4 (0.7) ^b^	<0.001 ^†^
**Nutrient consumption/day**				
Kilocalories	2238 (436) ^a^	2007 (264) ^a^	2442 (229) ^b^	<0.001 ^†^
Total fat (g)	79.5 (14.6) ^a^	70.3 (12.5) ^b^	96.9 (44.0) ^c^	<0.001 ^†^
Saturated fat (g)	31.3 (9.2)	28.5 (8.3) ^a^	36.4 (20.1) ^b^	0.015 ^†^
Monounsaturated fat (g)	26.7 (5.1) ^a^	25.5 (3.5) ^a^	30.0 (1.7) ^b^	<0.001 ^†^
Polyunsaturated fat (g)	16.3 (1.6) ^a^	15.4 (1.5) ^b^	15.7 (1.8) ^c^	0.004 ^†^
Total carbohydrates (g)	294 (50) ^a^	276 (57) ^a^	329 (36) ^b^	<0.001 ^†^
Complex carbohydrates (g)	232 (58) ^a^	222 (51) ^a^	263 (28) ^b^	<0.001 ^†^
Fiber (g)	14 ± 2 ^a^	14 ± 3 ^a^	16 ± 1 ^b^	0.001 *
**Physical activity**				
METs/day	61.5 (15.4)	61.1 (10.0)	62.8 (14.7)	0.88 ^†^
MVPA Blocks	1.3 (3.60)	1.3 (2.3)	1.0 (2.5)	0.317 ^†^
VPA Blocks	0.1 (1.0)	0.3 (1.0)	0.0 (1.3)	0.991 ^†^
Hours of TV/day	3.6 ± 1.7	3.8 ± 2.7	3.3 ± 3.1	0.57 *

OBMS: obesity with metabolic syndrome; OB: obesity; AW: Appropriate weight; BMI: Body Mass Index; HOMA: Homeostasis Model Assessment; HDL-C: high-density lipoprotein; LDL-C: low-density lipoprotein; hsCRP: high-sensitivity C-reactive protein; MET: Metabolic equivalent; MVPA: moderate to vigorous physical activity; VPA: vigorous physical activity; ^1^ Averages ± standard deviations and medians (interquartile ranges); ^2^ Different letters between lines show significant differences between groups; * ANOVA with Scheffé or ^†^ Kruskal-Wallis with Mann–Whitney U post-test.

In the TG fraction, the OBMS group has a significantly higher proportion of SFAs compared to the other groups (*p* = 0.001); additionally, the OB group had a higher proportion of SFAs compared to the AW (*p* = 0.028). Total MUFAs was significantly greater in the OBMS group compared to the AW group (*p* = 0.003). The proportion of palmitoleic-16:1*n*-9 for OBMS and OB was higher than AW (*p* = 0.009). Of the total PUFAs represented in this case by linoleic-18:2*n*-6, the OBMS group had the lowest proportion among the three groups (*p* = 0.001), followed by the OB group, with the highest being AW (*p* = 0.042) (Table 3). In this fraction, palmitic-16:0 correlated directly with the HOMA (*Rho* = 0.55 *p* = 0.001) and serum TG (*Rho* = 0.66 *p* = 0.001) and inversely with HDL-C (*Rho* = −0.52 *p* = 0.001). Linoleic-18:2*n*-6 was correlated inversely with the HOMA (*Rho* = −0.56 *p* = 0.001) and serum TG (*Rho* = −0.70 *p* = 0.001) and directly with HDL-C (*Rho* = 0.49 *p* = 0.001) (Table S1).

**Table 3:** Major fatty acids in lipid fractions of youth according to the study groups ^1^.

Triglycerides				
FA (% Total FA)	OBMS	OB	AW	*p* *^,†^
	*n* = 32	*n* = 32	*n* = 32	
**SFA sum**	38.3 (7.1) ^a, 2^	34.6 (8.9) ^b^	30.8 (7.6) ^c^	<0.001 ^†^
Palmitic-16:0	29.7 ± 4.0 ^a^	26.4 ± 3.7 ^b^	23.6 ± 3.3 ^c^	<0.001 *
Stearic-18:0	6.2 (3.8)	7.4 (4.1)	7.2 (3.8)	0.42 ^†^
**MUFA sum**	36.1 ± 5.2 ^a^	33.5 ± 5.0	31.7 ± 5.0 ^b^	0.003 *
Palmitoleic-16:1*n*-7 ^3^	4.5 (2.0) ^a^	4.1 (1.4) ^a^	3.0 (1.6) ^b^	0.009 ^†^
Oleic-18:1*n*-9	32.1 ± 4.3	30.7 ± 3.9	30.1 ± 4.3	0.14 *
**PUFA sum**	-	-	-	-
Linoleic-18:2*n*-6	25.6 ± 5.8 ^a^	32.2 ± 6.3 ^b^	36.4 ± 7.5 ^c^	<0.001 ^†^
**Phospholipids**				
**SFA sum**	52.8 (11.3) ^a^	54.1 (11.3)	56.4 (5.9) ^b^	0.017 ^†^
Palmitic-16:0	30.6 ± 3.4 ^a^	31.7 ± 3.0	33.2 ± 3.0 ^b^	0.006 *
Stearic-18:0	19.8 (4.9)	20.1 (6.0)	21.2 (2.1)	0.34 ^†^
Behenic-22:0	0.5 (0.2) ^a^	0.6 (0.2) ^b,c^	0.8 (0.2) ^b,c^	0.015 ^†^
**MUFA sum**	17.5 (7.3) ^a^	15.1 (8.3) ^b,c^	13.8 (3.5) ^b,c^	<0.001 ^†^
Palmitoleic-16:1*n*-7 ^4^	0.9 (0.4) ^a^	0.8 (0.2) ^b^	0.5 (0.2) ^c^	<0.001 ^†^
Oleic-18:1*n*-9	16.7 (7.3) ^a^	14.1 (8.1) ^b,c^	13.5 (3.4) ^b,c^	<0.001 ^†^
**PUFA sum**	30.0 (4.8)	31.1 (3.8)	29.9 (4.1)	0.74 ^†^
Linoleic-18:2*n*-6	18.0 ± 2.2 ^a^	19.2 ± 2.6 ^a^	21.0 ± 2.8 ^b^	<0.001 *
DHGL-20:3*n*-6	2.8 ± 0.7 ^a^	2.5 ± 0.4	2.1 ± 0.5 ^b^	<0.001 *
Eicosatrienoic-20:3*n*-3 ^5^	8.2 ± 2.2	7.7 ± 2.0	5.6 ± 2.7	0.063 *
Arachidonic-20:4*n*6	5.0 (1.2)	5.3 (1.5)	4.8 (2.3)	0.77 *
**Cholesterol Esters**				
**SFA sum**	35.7 (15.5)	35.1 (16.0)	35.7 (13.2)	0.92 ^†^
Palmitic-16:0	33.2 ± 7.7	31.0 ± 7.1	31.2 ± 5.2	0.35 *
Stearic-18:0 ^6^	10.4 (12.1)	11.3 (7.0)	12.3 (9.3)	0.96 ^†^
**MUFA sum**	-	-	-	-
Oleic-18:1*n*-9	28.4 (4.1)	26.3 (8.2)	26.2 (5.7)	0.44 ^†^
**PUFA sum**	-	-	-	-
Linoleic-18:2*n*-6	35.9 ± 9.5	34.5 ± 10.7	35.2 ± 10.4	0.84 *
**Free Fatty Acids**				
**SFA sum**	44.7 (4.1)	46.1 (6.4)	43.2 (6.7)	0.59 ^†^
Myristic-14:0	1.1 (0.2)	1.4 (0.1)	1.1 (0.5)	0.47 *
Palmitic-16:0	30.0 (2.4)	30.0 (3.7)	30.8 (4.5)	0.93 ^†^
Stearic-18:0	14.4 (2.2)	15.4 (2.7)	14.5 (3.4)	0.45 ^†^
**MUFA sum**	20.9 (4.1)	19.1 (4.6)	19.1 (5.6)	0.22 ^†^
Palmitoleic-16:1*n*-7 ^7^	1.7 (0.7)	1.7 (0.5)	1.5 (0.6)	0.46 ^†^
Oleic-18:1*n*-9	19.3 (4.0)	17.9 (3.3)	18.4 (3.4)	0.31 ^†^
**PUFA sum**	33.5 (3.4)	34.0 (4.1)	34.4 (4.9)	0.68 ^†^
Linoleic-18:2*n*-6	21.7 (3.3)	22.6 (3.3)	24.5 (5.3)	0.14 ^†^
DHGL-20:3*n*-6	3.0 ± 0.6 ^a^	2.8 ± 0.6	2.4 ± 2.5 ^b^	<0.001 *
Arachidonic-20:4*n*6	6.6 (1.1)	6.5 (3.1)	5.8 (2.0)	0.25 ^†^
DHA-22:6*n*-3	1.9 ± 0.4	1.9 ± 0.5	1.8 ± 0.5	0.51 *
**Total FFA** (mg/dL)	199.5 (40.0) ^a^	100.8 (24.1) ^b^	98.8 (29.9) ^b^	0.014 ^†^

OBMS: obesity with metabolic syndrome; OB: obesity; AW: Appropriate weight; SFA: saturated fatty acid; MUFA: monounsaturated fatty acid; PUFA: polyunsaturated fatty acid; FFA: free fatty acid; DHGL: Dihomo-gamma-linolenic; DHA: docosahexaenoic acid; * ANOVA; ^†^ Kruskal-Wallis test; ^1^ Data expressed as Averages±standard deviations or medians (interquartile ranges); ^2^ Different letters between lines show significant differences between groups, according to the Scheffé post-test for parametric variables or the Mann–Whitney U test for nonparametric variables; ^3^ OBMS *n* = 28, OB *n* = 20, AW *n* = 16; ^4^ OBMS *n* = 28 OB *n* = 25, AW *n* = 8; ^5^ OBMS *n* = 15, OB *n* = 13, AW *n* = 6; ^6^ OBMS *n* = 15, OB *n* = 13, AW *n* = 6; ^7^ OBMS *n* = 27, OB *n* = 19; AW *n* = 8.

In the PL fraction, total SFAs were present in significantly lower amounts in the OBMS group compared to the AW group. The OBMS group had higher total MUFAs compared to the other two groups (*p* = 0.001), with no difference between the OB and AW groups (*p* = 0.05). In particular, palmitoleic-16:1*n*-7 was higher in OBMS compared to the other two groups (*p* = 0.001), and time was higher in OB than AW (*p* = 0.004). With respect to linoleic-18:2*n*-6, a significantly lower proportion was found in the OBMS and the OB group compared to the AW group (*p* = 0.001, *p* = 0.021, respectively). DHGL-20:3*n*-6 had a significantly higher proportion in the OBMS group compared to the AW group (*p* = 0.001), with no differences between OB and AW (*p* = 0.079) (Table 3). In the PL fraction, palmitoleic-16:1*n*-7 was directly associated with serum TG (*Rho* = 0.54 *p* = 0.001) and inversely associated with HDL-C (*Rho* = −0.43 *p* = 0.001). DHGL-20:3*n*-6 correlated directly with the HOMA (*Rho* = 0.36 *p* = 0.000) and serum TG (*Rho* = 0.33 *p* = 0.001), while linoleic-18:2*n*-6 correlated inversely with the HOMA (*Rho* = −0.31 *p* = 0.002) and serum TG (*Rho* = −0.29 *p* = 0.004) and directly with HDL-C (*Rho* = 0.30 *p* = 0.003) (Table S1).

In the FFA fraction, total circulating FFAs were significantly greater in the OBMS group, with double the concentration compared to the other groups (*p* = 0.014). The OB group had the same concentration of total FFAs as the AW group (Table 3). Among PUFAs, DHGL-20:3*n*-6 was significantly higher in the OBMS group than in the AW group (*p* = 0.001); with no differences between OB and AW. The DHGL-20:3*n*-6/linoleic-18:2*n*-6 ratio was significantly greater in the OBMS and OB groups compared to the AW group (*p* = 0.001) (Table 4). DHGL-20:3*n*-6 correlated directly with the HOMA (*Rho* = 0.36 *p* = 0.001) and TG (*Rho* = 0.33 *p* = 0.001) (Table S1).

**Table 4:** Ratios of fatty acids in lipid fractions of youth according to the study groups ^1^.

Ratio of Fatty Acids	OBSM	OB	AW	*p* *^,†^
	*n* = 32	*n* = 32	*n* = 32	
Triglycerides				
16:1/16:0 ^3^	0.1 (0.1) ^a, 2^	0.1 (0.2)	0.1 (0.1) ^b^	0.005 ^†^
18:1/18:0	5.1 ± 1.9	4.4 ± 1.9	4.4 ± 1.8	0.30 ^†^
**Phospholipids**				
16:1*n*-7/16:0 ^4^	0.03 (0.01) ^a^	0.02 (0.01) ^b^	0.01 (0.01) ^c^	<0.001 ^†^
18:1*n*-9/18:0	0.8 (0.7) ^a^	0.7 (0.7)	0.6 (0.2) ^b^	<0.001 ^†^
20:3*n*-6/18:2*n*-6	0.2 (0.1) ^a^	0.1 (0.0) ^b^	0.1 (0.0) ^c^	<0.001 ^†^
20:4*n*6/20:3*n*6	1.9 (0.7)	2.0 (1.2)	2.3 (1.4)	0,059 ^†^
**Cholesterol Esters**				
18:1*n*-9/18:0 ^5^	2.3 (2.6)	2.4 (1.7)	2.1 (1.6)	0.94 *
**Free Fatty Acids**				
16:1/16:0 ^6^	0.5 (0.0)	0.6 (0.0)	0.5 (0.0)	0.57 ^†^
18:1*n*-9/18:0	1.3 (0.4)	1.2 (0.4)	1.3 (0.4)	0.47 ^†^
20:3*n*-6/18:2*n*-6	0.1 ± 0.0 ^a^	0.1 ± 0.0 ^a^	0.1 ± 0.0 ^b^	<0.001 *
20:4*n*6/20:3*n*6	2.2 (0.8)	2.3 (0.8)	2.5 (1.5)	0.22 ^†^

OBMS: obesity with metabolic syndrome; OB: obesity; AW: Appropriate weight; * ANOVA; ^†^ Kruskal-Wallis test; ^1^ Data expressed as Averages±standard deviations, medians (interquartile ranges); ^2^ Different letters between lines show significant differences between groups, according to the Scheffé post-test for parametric variables or the Mann–Whitney U test for nonparametric variables; ^3^ OBMS *n* = 28, OB *n* = 20, AW *n* = 16; ^4^ OBMS *n* = 28, OB *n* = 25, AW *n* = 18; ^5^ OBMS *n* = 15, OB *n* = 13, AW *n* = 6; ^6^ OBMS *n* = 27, OB *n* = 19, AW *n* = 8.

A descriptive multivariate analysis of the HOMA revealed that WC increased the average HOMA by 1.025, total FFAs increased average HOMA by 1.004, and each unit of DHGL-20:3*n*-6 in FFAs increased the average HOMA by 1.258, while physical activity (METs) and linoleic-18:2*n*-6 in TG decreased average HOMA by −1.018 and −1.016, respectively. The independent variables explained 62% of the behavior of the HOMA. All relationships observed were significant and remained the same after making adjustments for food consumption variables (Table 5).

**Table 5:** Multiple linear regression model with explanatory variables of the HOMA index.

Variables	β *	β ^†^	IC ^†^	*p* ^†^	IFV ^†^
Waist circumference	1.025	1.025	0.008; 0.015	0.000	1.543
Average METs/d	−1.018	−1.018	−0.012; −0.003	0.001	1.278
Linoleic-18:2*n*-6 in TG	−1.016	−1.016	−0.013; −0.001	0.024	1.606
DHGL-20:3*n*-6 in FFA	1.276	1.258	0.028; 0.172	0.007	1.767
Total FFA in mg/dL	1.004	1.004	0.000; 0.003	0.028	1.379

HOMA: The Homeostatic model assessment.* Raw model: *R*^2^: 0.637, ANOVA: *p* = 0.0001, Normality: *p* = 0.200, Colinearity: Inflation factor of the variance (IFV) <5, Durbin Watson: 0.930. Values expressed in HOMA units. ^†^ Adjusted for total calories, saturated fat, monounsaturated fat, polyunsaturated fat and simple carbohydrates. Values expressed in HOMA units. Adjusted model: *R*^2^: 0.620, ANOVA: 0.0001, Normality: *p* = 0.050, Colinearity: IFV < 5, Durbin Watson: 0.987.

## 4. Discussion

This study detected proportions of some FA for youth with OBMS in the TG, PL and FFA fractions that correlated with disturbances of MetS. 

In OBMS the TG fraction showed significantly higher concentrations of total SFAs and MUFAs, but a lower concentration of PUFAs. This pattern could be due to accelerated hepatic lipogenesis that results in the incorporation of FAs, such as palmitic-16:0, palmitoleic-16:1*n*-7 and oleic-18:1*n*-9 in VLDL, with a simultaneous decrease in the amount of linoleic-18:2*n*-6 [19]. 

This study, consistent with that detected previously by others, found high proportions of MUFAs in the OBMS and OB groups [23,24,49]. Palmitoleic-16:1n-7 was directly and significantly associated with serum TG. The palmitoleic-16:1*n*-7/palmitic-16:0 and oleic-18:1*n*-9/stearic-18:0 ratios that could indirectly indicate enzyme activity of delta-9 desaturase were higher in the OBMS group, consistent with other studies [10,11,12,24] where they have been associated with obesity [10], IR and MetS [12]. The increase in the activity levels of desaturase enzymes in obesity could be related to hyperinsulinemia arising from excess states, as insulin is a potent activator and regulator of delta-6 and delta-9 desaturases [50]. 

This study found low proportions of PUFAs in the OBMS group. The concentrations of linoleic-18:2*n*-6 in both the TG and PL fractions were correlated directly with HDL-C and inversely with serum TG and the HOMA. These inverse relationships could be explained because reduced availability of PUFAs in cell membranes has been associated with lower fluidity and changes in insulin sensitivity [51]. 

DHGL-20:3*n*-6, a product of desaturation and elongation of linoleic-18:2n-6, appears at significantly higher percentages in the PL and FFA fractions in the OBMS group than in the AW group; consistent with this finding, the DHGL-20:3*n*-6/linoleic-18:*n*-6 ratio was higher in the OBMS group in both fractions, with similar results as those reported by Okada [11]. DHGL-20:3*n*-6 in the PL and FFA fractions correlated positively and significantly with WC, HOMA and TG, which agrees with other studies that conclude that this FA is associated with greater IR, cardiovascular risk [10,22,24,25] and inflammation [22]. In adults, Kurotani et al. also associated this FA and the DHGL-20:3*n-*6/linoleic-18:2*n-*6 ratio with early markers of IR and DM, such as high levels of C peptide [52]. DHGL-20:3*n-*6 is a precursor of thromboxanes and series 1 prostanoids (anti-inflammatory) via cyclooxygenase; its increase in these young people, without a simultaneous increase in ARA-20:4*n*-6, could indicate a compensatory mechanism associated with increased activity of delta-6 desaturase, which would enhance the anti-inflammatory activity helping to maintain the balance between pro- and anti-inflammatory substances [53]. 

The main finding in the FFA fraction was that the OBMS group had double the circulating total FFAs of other groups. This finding has been reported by other authors [54,55], in addition to its positive correlations with WC, insulin, HOMA [55], CT and serum TG [54]. The increase in total FFAs suggests that in those who present central obesity, adipocytes increase FA release due to saturation of the storage capacity and stress to the endoplasmic reticulum of visceral adipocytes. This effect diminishes the anti-lipolytic signal of insulin, perpetuating the release of FAs from visceral adipose tissue, which, once in circulation, are lipotoxic and are associated with the development of lipid alterations, IR and MetS [6,55,56].

Given the aforementioned associations, it was investigated whether FAs could explain the link between visceral obesity and insulin resistance in these youth using a multiple linear regression model. It was found that 62% of the HOMA was explained by the increases in total FFAs, DHGL-20:3*n*-6 in FFA and WC, in addition to decreases in linoleic-18:2*n*-6 in TG and the average METs/d. 

The OB group presented proportions similar to those of the AW group in DHGL-20:4*n*-6 and in the total amount of circulating FFAs, variables – which in the OBMS group – has been associated with metabolic alterations, especially with IR. However, the OB group, compared to the AW group, presented a greater proportion of SFAs and MUFAs, a lower proportion of PUFAs in the TG fraction, higher values of WC, HOMA and TG and lower proportions of HDL-C, which is consistent with increased lipogenesis and confirms that obesity itself is a disorder that leads to future metabolic abnormalities and probably to NAFLD, if weight gain continues; consequently, the OB youth group should not be considered metabolically healthy. In these obese youth, therapeutic measures necessary to avoid or diminish MetS and NAFLD should be considered, especially in stimulating weight reduction with an increase in physical activity and the adoption of healthy eating habits, among others, as well as encouraging a greater consumption of polyunsaturated fat, a source of Omega 3 FA; if necessary, prescribe the consumption of lipid-lowering agents [57]. 

Like Waresnjo, this study found no association between circulating FAs and the PA level [12]; however, PA was incorporated as an explanatory variable of the HOMA in the multiple regression model, indicating that certain FAs in the profile and environmental factors influence its value. 

In this study, the AW group consumed more calories and fats than the obese groups, results that have been reported by other authors [58,59]; these findings may be due to underestimations in evaluations of self-reported consumption, especially in obese individuals, or, when excess weight is evident, obese individuals decrease food intake. Therefore, current consumption cannot be correlated with nutritional status. 

We did not detect a consumption pattern that could explain the differences in the FA profiles among the groups. Qualitative analysis of diets allowed us to establish that these youth did not consume significant omega-3 food sources, such as fish or seed oils, which could explain to some degree the low proportions of these FAs in the different fractions. In any event, to eliminate the effect of consumption on the descriptive model of the HOMA index, an adjustment was made for these variables, and the model was not modified. 

This study has some limitations: the 24-h recall does not allow establishing individual associations with biochemical variables because of intra-individual variations in food intake; estimation of desaturase enzymes was performed using an indirect method due to technical difficulties involving their measurement *in vivo*. The diagnostic criteria for NAFLD in youth were not evaluated, which prevents establishing some degree of association with the profile of the fatty acids of the different fractions.

Finally, it is expected that there will be further research involving more complex lipids, such as waxes and eicosanoids, and methodological designs that establish causal relationships between the FA profile and metabolic disturbances that make up MetS, especially in children and youths.

## 5. Conclusions

This study detected proportions of some FA for youth with OBMS in the TG, PL and FFA fractions that correlated with disturbances of MetS, principally IR. High concentrations of total FFAs, high proportions of palmitic-16:0 in TG, high palmitoleic-16:1*n*-7 and low linoleic-18:2*n*-6 in TG and PL, and high DHGL-20:2*n*-6 in PL and FFAs could all be early markers of IR in youths. The findings further suggest that the connection between FA profiles and metabolic disturbances is associated more with high WC than BMI. The OB group presented greater proportions of SFA and MUFA and lower proportions of PUFA, as well as some higher biochemical values than those youth with AW, suggesting the possibility of developing future metabolic abnormalities if weight gain continues.

## Supplementary Materials

The following are available online at http://www.mdpi.com/2072-6643/8/2/54/s1, Table S1 Correlations between fatty acids of triglycerides, phospholipids, free fatty acids fractions, and cardiovascular risk factors in adolescents.

## Abbreviations

ARA: arachidonic acid
AW: appropriate weight
BMI: Body Mass Index
CE: cholesterol ester
HDL-C: high-density lipoprotein
LDL-C: low-density lipoprotein
DHA: docosahexaenoic acid
DHGL: dihomo-γ-linolenic acid
DM: diabetes mellitus
FA: fatty acid
FFA: free fatty acid
HOMA: Homeostasis Model Assessment
hsCRP: high-sensitivity *C*-reactive protein
HT: hypertension
IR: insulin resistance
MET: Metabolic equivalent
MetS: metabolic syndrome
MUFA: monounsaturated fatty acid
MVPA: moderate to vigorous physical activity
OBMS: obese with metabolic syndrome
OB: obese
PA: physical activity
PL: phospholipid
PUFA: polyunsaturated fatty acid
SFA: saturated fatty acid
TG: triglyceride
VLDL: very-low-density lipoprotein
VPA: vigorous physical activity
WC: waist circumference
