# Supplementary material for: Profile of Free Fatty Acids and Fractions of Phospholipids, Cholesterol Esters and Triglycerides in Serum of Obese Youth with and without Metabolic Syndrome
Source: Nutrients. 2016 Feb 15;8(2):54. doi: 10.3390/nu8020054 (PMC4772025; doi:10.3390/nu8020054)
Supplement: Supplementary file 1 [file nutrients-08-00054-s001.docx]

**Supplementary Materials: Profile of Free Fatty Acids and Fractions of Phospholipids, Cholesterol Esters and Triglycerides in Serum of Obese Youth with and without Metabolic Syndrome**

**Juliana Bermúdez-Cardona and Claudia Velásquez-Rodríguez**

**Table S1.** Correlations between fatty acids of triglycerides, phospholipids, free fatty acids fractions, and cardiovascular risk factors in adolescents ^1^.

| **Fatty Acid** | **BMI** | **WC** | **SFF** | **TFF** | **% Fat** | **Insulin** | **HOMA** | **TG** | **HDL-C** | **hsPCR** |
| --- | --- | --- | --- | --- | --- | --- | --- | --- | --- | --- |
| **Triglycerides** | | | | | | | | | | |
| Palmitic-16:0 | 0.453 * | 0.474 * | 0.433 * | 0.366 * | 0.387 | 0.558 | 0.557 | 0.667 | −0.528 | 0.290 |
| Palmitoleic-16:1*n-*7 | 0.369 | 0.289 | 0.360 | 0.349 | 0.364 | NS | NS | 0.362 | 0.262 | 0.339 |
| Linoleic-18:2*n-*6 | −0.440 * | −0.456 * | −0.395 * | −0.362 * | −0.335 | −0.563 | −0.561 | −0.700 | 0.494 | −0.262 |
| **Phospholipids** | | | | | | | | | | |
| Palmitic-16:0 | −0.269 * | −0.261 * | NS * | NS | NS | −0.368 | −0.312 | NS | NS | NS |
| Palmitoleic-16:1*n*-7 | 0.357 | 0.347 | 0.334 | 0.346 | 0.282 | 0.321 | 0.322 | 0.541 | −0.436 | 0.294 |
| Oleic-18:1*n*-9 | 0.328 | 0.282 | 0.225 | 0.255 | 0.212 | 0.366 | 0.364 | 0.262 | −0.277 | 0.264 |
| Linoleic-18:2*n*-6 | −0.386 * | −0.369 * | −0.241 * | −0.302 * | −0.329 | −0.314 | −0.310 | −0.295 | 0.304 | −0.308 |
| DHGL-20:3*n*-6 | 0.292 * | 0.329 * | 0.214 * | 0.241 * | 0.200 | 0.361 | 0.363 | 0.334 | NS | NS |
| **Free Fatty Acids** | | | | | | | | | | |
| Total FFA | 0.276 | 0.231 | NS | NS | NS | 0.276 | 0.278 | −0.206 | NS | NS |
| Palmitoleic-16:1*n*-7 | 0.336 | 0.321 | 0.307 | 0.276 | 0.291 | NS | NS | NS | NS | NS |
| DHGL-20:3*n*-6 | 0.310^2^ | 0.311 | 0.262 | 0.253 | 0.219 | 0.372 | 0.369 | 0.333 | NS | NS |

Numeric values are significant at a value of <0.05. NS: not significant; BMI: Body Mass Index; WC: waist circumference; SFF: Subscapular fat fold; TFF: Triceps fat fold; % Fat: Percentage of fat; HOMA: homeostatic model assessment; TG: Triglycerides; HDL-C: high-density lipoprotein; hsCRP: high-sensitivity *C*-reactive protein; ^1^ The majority of the values corresponds to Spearman’s correlation, unless indicated to the contrary with an (*); * Pearson’s correlation; Palmitic-16:0 showed a moderate and significant association with all the anthropometric variables and some biochemicals such as insulin, HOMA and TG.
Inverse associations were observed with HDL-C in the triglycerides (TG) fraction. Palmitoleic-16:1*n*7 showed a positive correlation with the anthropometric variables, which is consistent in TG, PL and free fatty-acids (FFA) fractions. Although low, the value of the correlations is statistically significant, which relates this FA to adiposity and lipogenesis. With respect to the biochemical variables, in PL and TG, the palmitoleic-16:1*n*7 correlated positively with TG and inversely with HDL; in the PL fraction, it also correlated positively with insulin and HOMA, which relates this FA to RI and MetS. DHGL-20:3*n*6 correlated positively with the anthropometric variables (WC, SFF, TFF, BMI and % fat) in the PL and TG fractions; a positive correlation was found between DHGL-20:3*n*6 and the biochemical variables, the most important of which were insulin, HOMA and TG which relates this FA to RI and MetS. Linoleic-18:2*n*-6 associated inversely with the anthropometric variables of BMI, WC, SFF, TFF, % fat and weight, with greater strength in the biochemical variables, correlating negatively with insulin, HOMA and TG and positively with HDL-C, in the PL and TG fraction, which indicates that this FA acts as a protector in MetS.
